# Strategies to enhance monoclonal antibody uptake and distribution in solid tumors

**DOI:** 10.20892/j.issn.2095-3941.2020.0704

**Published:** 2021-08-15

**Authors:** Brandon M. Bordeau, Joseph P. Balthasar

**Affiliations:** 1 Department of Pharmaceutical Science, University at Buffalo, Buffalo, NY 14214, USA

**Keywords:** Solid tumors, antibody uptake and distribution, monoclonal antibody, antibody–drug conjugate

## Abstract

Despite the significant resources dedicated to the development of monoclonal antibody (mAb) therapies for solid tumors, the clinical success, thus far, has been modest. Limited efficacy of mAb in solid tumors likely relates to unique aspects of tumor physiology. Solid tumors have an aberrant vasculature and a dense extracellular matrix that slow both the convective and diffusive transport of mAbs into and within tumors. For mAbs that are directed against cellular antigens, high antigen expression and rapid antigen turnover can result in perivascular cells binding to and eliminating a significant amount of extravasated mAb, limiting mAb distribution to portions of the tumor that are distant from functional vessels. Many preclinical investigations have reported strategies to improve mAb uptake and distribution; however, to our knowledge, none have translated into the clinic. Here, we provide an overview of several barriers in solid tumors that limit mAb uptake and distribution and discuss approaches that have been utilized to overcome these barriers in preclinical studies.

## Introduction

In 1900, Paul Ehrlich developed the receptor theory, which was built on the foundational hypothesis that toxins, nutrients, and drugs exert their observed effect through binding to unique proteins that are present within cells^1^. As a natural consequence of receptor theory, Ehrlich believed that drugs could be developed that can specifically bind to and neutralize a disease-causing organism while sparring the host cells^1^. Ehrlich famously developed the term “magic bullet” to describe the mechanism by which cells developed immunity to toxins and was inspired to apply this concept to the development of small-molecule drugs with the postulate “we have to learn how to aim chemically”^1^. Ehrlich is considered to be the founder of modern chemotherapy; his research led to the development of Salvarsan as a treatment for syphilis, and his many theories were foundational to the development of the first anti-cancer drugs^1^.

Currently, monoclonal antibodies (mAbs) are heralded as the “magic bullets” that Ehrlich envisioned, and in many cases, the moniker is well deserved. Antibodies can bind most substances with high affinity and high selectivity and are used for the treatment of many diseases. A case in which mAbs do not live up to their hyperbolic nickname is as therapies directed against solid tumors. Although 18 mAbs are approved for solid-tumor indications, it is generally accepted that the observed efficacy is disappointing^2–5^. **Table 1** provides a list of all Food and Drug Administration (FDA)–approved mAbs for solid tumor indications and the clinical trial outcomes that resulted in approval. On average, for mAbs that were investigated in 2-arm clinical trials, an increase in progression-free survival of 3 months and a 10.3% increase in the objective response rate were observed in the mAb treatment arm. Despite the marginal benefit, the cost of mAb therapy for oncology is high, with a median cost of $142,833 per year^6^. Currently, the clinical methods that are being used to improve mAb efficacy focus on improving patient selection through genomic or proteomic screening prior to mAb therapy^7–9^. For example, trastuzumab requires human epidermal growth factor receptor 2 (HER2) screening with an immunohistochemical or fluorescence *in situ* hybridization assay to ensure HER2 overexpression prior to therapy^7^. However, in many cases, the assessment of target expression alone is not sufficient to predict a patient’s response to mAb therapy^10–12^.

**Table 1:** FDA approved mAbs for solid tumor indications

Antibody (mAb)	Format	Indication	Target	Year approved	Trial	Trial arm	Comparator	Notes
Trastuzumab (Herceptin)	Humanized IgG1 mAb	Breast cancer	HER2	1998	mAb + chemotherapy *vs.* chemotherapy	MTP: 7.2 months ORR: 45% DOR: 8.3 months 1-year survival: 79%	MTP: 4.5 months ORR: 29% DOR: 5.8 months 1-year survival: 68%	Approved in combination with paclitaxel for patients without prior chemotherapy or as a single agent in patients who have progressed on chemotherapy
Cetuximab (Erbitux)	Chimeric IgG1 mAb	Colorectal cancer	EGFR	2004	mAb + irinotecan *vs.* mAb	ORR: 22.9% MTP: 4.1 months	ORR: 10.8% MTP: 1.5 months	Assessed in patients who progressed on irinotecan. Approved in combination with irinotecan for patient’s refractory to irinotecan or as a single agent for patients intolerant to irinotecan
Bevacizumab (Avastin)	Humanized IgG1	Colorectal cancer	VEGF	2004	mAb + IFL *vs.* IFL + placebo	MTP: 20.3 months PFS: 10.6 months ORR: 45% DOR: 10.4 months	MTP: 15.6 months PFS: 6.4 months ORR: 35% DOR: 7.1 months	Tested in combination with 5-fluorouracil, irinotecan, leucovorin
Panitumumab (Vectibix)	Humanized IgG2 mAb	Colorectal cancer	EGFR	2006	mAb + best supportive care *vs.* best supportive care	PFS: 96 days ORR: 8%	PFS: 60 days ORR: 0%	Assessed in patients who progressed on fluoropyrimidine, oxaliplatin, and irinotecan. No difference in overall survival observed
Pertuzumab (Perjeta)	Humanized IgG1	Breast cancer	HER2	2012	mAb + trastuzumb + docetaxel *vs.* placebo + trastuzumab + docetaxel	PFS: 18.5 months ORR: 80.2% DOR: 20.2 months Survival: 82.8%	PFS: 12.4 months ORR: 69.3% DOR: 12.5 months Survival: 76.4%	Approved in combination with trastuzumab and docetaxel for patients who have not received prior therapy
Ado-trastuzumab emtansine (Kadcyla)	Humanized IgG1 ADC	Breast cancer	HER2	2012	ADC *vs.* lapatinib + capecitabine	PFS: 9.6 months ORR: 43.6% DOR: 12.6 months MS: 30.9 months	PFS: 6.4 months ORR: 30.8% DOR: 6.5 months MS: 25.1 months	Tested in patients with metastatic or locally advanced breast cancer with prior trastuzumab or prior taxane therapy
Ramucirumab (Cyramza)	Human IgG1	Gastric cancer	VEGFR2	2014	mAb + best supportive care *vs.* best supportive care + placebo	PFS: 2.1 months MS: 5.2 months	PFS: 1.3 months MS: 3.8 months	Tested in patients with locally advanced or metastatic gastric cancer who previously received platinum or fluoropyrimidine chemo
Nivolumab (Opdivo)	Human IgG4	Melanoma/ NSCLC	PD1	2014	Single-arm trial	ORR: 32%	N/A	Tested in patients with unresectable or metastatic melanoma that progressed on ipilimumab
Pembrolizumab (Keytruda)	Humanized IgG4	Melanoma	PD1	2014	Single-arm trial	ORR: 24%	N/A	Tested in patients with unresectable or metastatic melanoma that progressed on ipilimumab
Necitumumab (Portrazza)	Human IgG1	NSCLC	EGFR	2015	mAb + gemcitabine + cisplatin *vs.* gemcitabine + cisplatin	PFS: 5.7 months OS: 23%ORR: 31% MS: 11.5 months	PFS: 5.5 months OS: 19%ORR: 29% MS: 9.9 months	Tested as a first line chemotherapy in patients with metastatic squamous NSCLC
Dinutuximab (Unituxin)	Chimeric IgG1	Neuroblastoma	GD2	2015	mAb + RA *vs.* RA	OS: 73% EFS: 71%	OS: 58% EFS: 56%	Tested in pediatric patients with high risk neuroblastoma. “RA = 13-*cis*-retinoic acid”
Olaratumab (Lartruvo)	Human IgG1	Soft tissue sarcoma	PDGFR-alpha	2016	mAb + doxorubicin *vs.* doxorubicin	OS: 41% MS: 26.5 months PFS: 8.2 months ORR: 18.2%	OS: 22% MS: 14.7 months PFS: 4.4 months ORR: 7.5%	Eligible patients were required to have soft tissue sarcoma not amenable to curative treatment with surgery or radiotherapy, a histologic type of sarcoma for which an anthracycline-containing regimen was appropriate but had not been administered
Atezolizumab (Tecentriq)	Humanized IgG1	Bladder cancer	PD-L1	2016	Single-arm trial	ORR: 14.8%	N/A	Tested in patients with locally advanced or metastatic urothelial carcinoma that progressed on platinum containing chemotherapy.
Avelumab (Bavencio)	Human IgG1	Merkel cell carcinoma	PD-L1	2017	Single-arm trial	ORR: 33%	N/A	Tested in patients who progressed on chemotherapy for distant metastatic disease
Durvalumab (IMFINZI)	Human IgG1	Bladder cancer	PD-L1	2017	Single-arm trial	ORR: 17%	N/A	Tested in patients with metastatic urothelial cancer that progressed on or after a platinum-based therapy
Cemiplimab (Libtayo)	Human mAb	Cutaneous squamous cell carcinoma	PD-1	2018	Single-arm trial	ORR: 47.2%	N/A	Tested in patients with metastatic or locally advanced CSCC that were not candidates for curative surgery or radiation
Enfortumab vedotin (Padcev)	Human IgG1 ADC	Urothelial cancer	Nectin-4	2019	Single-arm trial	ORR: 44% DOR: 7.6 months	N/A	Tested in patients with locally advanced or metastatic urothelial cancer with prior PD-1 or platinum-based chemo
[fam-]trastuzumab deruxtecan-nxki (Enhertu)	Humanized IgG1 ADC	Metastatic breast cancer	HER2	2019	Single-arm trial	ORR: 60.3% DOR: 14.8 M	N/A	Tested in patients with HER2-positive, unresectable and/or metastatic breast cancer who had received 2 or more prior anti-HER2 therapies
Sacituzumab govitecan-hziy (Trodelvy)	Humanized IgG1 ADC	Triple-neg. breast cancer	TROP-2	2020	Single-arm trial	ORR: 33.3% DOR: 7.7 M	N/A	Tested in patients with metastatic triple negative breast cancer who progressed on 2 prior treatments.

mAb, antibody; ADC, antibody drug conjugate; OS, overall survival; PFS, progression-free survival; ORR, objective response rate; MS, median survival; DOR, median duration of response; EFS, event-free survival.

**Figure 1:**
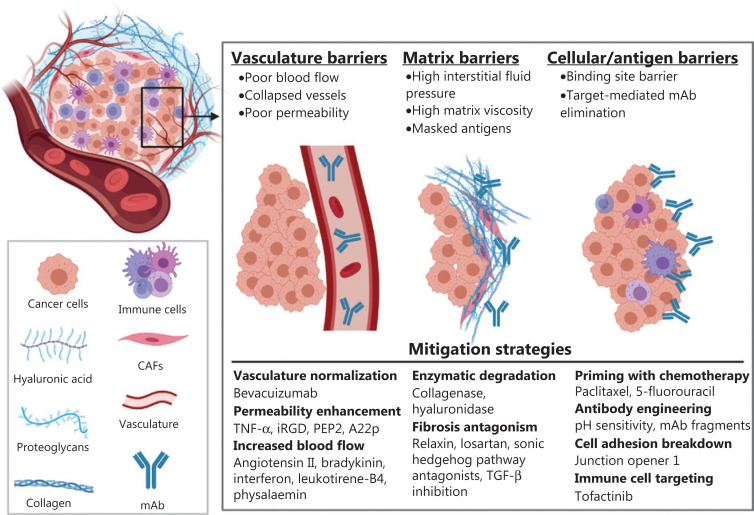
Shown is a graphic representation of barriers that limit therapeutic antibody uptake and distribution into solid tumors and the approaches that have been reported to mitigate the tumor barriers. A graphic key is provided in the bottom left inset. Figure 1 was created using BioRender.com.

Antibodies exert therapeutic effects through a variety of mechanisms. Antibodies may antagonize tumor growth pathways by binding to cell membrane-associated receptors, inhibiting their activation or enhancing their degradation or by binding to and neutralizing soluble growth factors^13^. Antibodies that bind cell membrane receptors can also recruit immune effector cells through fragment crystallizable (Fc) gamma receptor binding, leading to tumor cell killing through antibody-dependent cellular cytotoxicity by natural killer (NK) cells or antibody-dependent cell phagocytosis by monocytes and macrophages^13,14^. Antibodies that bind to cell membrane receptors can also trigger complement-dependent cytotoxicity with tumor cell lysis resulting from the formation of the membrane attack complex and the recruitment of NK cells, macrophages, neutrophils, dendritic cells, and T-cells following complement receptor stimulation^15^. Many of the anti-cancer mAbs recently approved by the FDA bind to the programmed death receptor or ligand 1 (PD-1), blocking a key mechanism of immune escape for many tumors^16^. Antibodies are also used for the delivery of cytotoxic drugs, radionuclides, and immunotoxins^13^. A common barrier that limits the efficacy of all mAb-based therapies is poor uptake and distribution within solid tumors, which results in suboptimal exposure to portions of the tumor, development of resistance, and tumor progression^2^. The abnormal physiology of solid tumors and its relevance to antibody delivery have been reviewed elsewhere^17–23^. Here, we provide a brief overview of the individual tumor barriers and focus on a discussion of strategies that have been explored to overcome each barrier for the enhancement of mAb tumor uptake and penetration (**Figure 1**).

## Vasculature

The blood vasculature within solid tumors is composed of many immature and disorganized vessels, resulting in poor blood flow and hypoxia^18,20,24,25^. The structural deficiencies of solid tumor blood vessels and the overexpression of pro-angiogenic factors that increase vasculature permeability result in plasma leakage into the interstitial space^25^. High interstitial oncotic pressure can collapse tumor blood vessels and can limit the convective transport of mAbs from the blood into tumor interstitial fluid^17,18,23^.

In solid tumors, the rate of mAb extravasation is much slower than the rates of interstitial diffusion and antigen binding^26^. As a result, many strategies have been explored to modulate tumor vasculature to improve mAb uptake (**Table 2**). The most notable approach to modify the vasculature in tumors is to modulate angiogenesis and vessel porosity with agents such as the anti-vascular endothelial growth factor (VEGF) mAb bevacizumab. Bevacizumab can “normalize” tumor vasculature by pruning immature and leaky vessels, improving blood flow, and decreasing interstitial fluid pressure (IFP)^24,25^. Enhanced blood flow and decreased IFP improves mAb tumor uptake; however, the window between normalization and excessive pruning is dependent on both the dose of the anti-angiogenic and the time after administration and has proven difficult to capture^24,25,27^. Our group observed a 63% decrease in the tumor area under the curve (AUC) up to 10 days after administration of the anti-carcinoembryonic antigen (CEA) mAb T84.66 in LS174T xenograft-bearing mice that were treated with 5 mg/kg bevacizumab twice a week^28^. Decreased tumor uptake with bevacizumab co-administration has also been observed with the anti-HER2 mAb trastuzumab in multiple xenograft model^29–31^. In a phase II trial, the combination of bevacizumab with trastuzumab and docetaxel did not improve patient survival^32^. Administration of 5 mg/kg bevacizumab to mice bearing OSC19 and SCC1 xenografts, 3 days prior to the administration of 10 mg/kg IRDye800 cetuximab, resulted in an increase in cetuximab tumor fluorescence^33^. A single 10-mg/kg dose of bevacizumab to mice bearing SUM149 xenografts, 4 days prior to the administration of 2.2 μg of indium-111 (^111^In)–radiolabeled mAb, decreased the tumor uptake of cetuximab by 40% and decreased the uptake of an anti-insulin growth factor 1 receptor mAb R1507 by 35%^34^. Bevacizumab has been evaluated in 2 phase III clinical trials with both cetuximab and panitumumab. The combination of bevacizumab with cetuximab did not significantly improve patient survival^35^, and the combination of panitumumab and bevacizumab decreased progression-free survival^36^. The disappointing clinical trial results for mAb–bevacizumab co-therapy are in contrast to the results obtained for chemotherapy–bevacizumab combinations^37–39^. The disconnect may result from the difference in the rate-limiting step for tumor uptake of small-molecule drugs (SMDs) in comparison to mAbs. SMDs have high vascular permeability and can rapidly diffuse into tumors from the blood. For tumor regions with poor blood flow, SMDs can enter the tumor space more quickly than the blood flow delivering the therapeutic agents (e.g., flow-limited distribution)^40^. Antibodies have much slower permeability rates than small molecules. As a result, tumor uptake of mAb is dependent on the permeability surface area product^40^. Improved tumor blood flow from vasculature normalization may enhance SMD uptake, while the decrease in tumor vasculature surface area, which results from the removal of immature tumor vessels, may contribute to the decrease in mAb tumor uptake observed with bevacizumab therapy.

**Table 2:** Vasculature modulation approaches

Treatment	Impact on tumor	Impact on mAb tumor PK	mAb/tumor model	Source
Bevacizumab	Decrease in tumor vasculature and permeability	63% decrease in AUC (0–10 days)	T84.66 LS174T	^28^
Sorafenib	Decrease in tumor vasculature and permeability	41% decrease in AUC (0–7 days)	T84.66 LS174T	^131^
Angiotensin II	Increase in trans vascular pressure gradient, enhanced tumor blood flow	40% increase in uptake 4 h after administration	CC49 LS174T	^41^
TNF-alpha administered intravenously (IV) or intra-tumorally (IT)	Enhanced vasopermeability	(IT) 200% increase at 3 h, 27% increase at 22 h (IV) 100% increase at 3 h	Mab35 LOVO	^42^
Interferon	Enhanced blood flow	83% increase in uptake 1.5 h after administration	MEM136 WM-9	^43^
Interleukin 2 conjugate	Enhanced vasopermeability	275% increase in uptake 3 days after administration	TNT-1 F(ab′)2 ME-180	^45^
TNF-alpha conjugate	Enhanced vasopermeability and blood flow	213% increase in uptake 3 days after administration	TNT-1 F(ab′)2 ME-180	^45^
Interleukin 1 conjugate	Enhanced vasopermeability and blood flow	200% increase in uptake 3 days after administration	TNT-1 F(ab′)2 ME-180	^45^
Leukotriene-B4 conjugate	Enhanced blood flow	122% increase in uptake 3 days after administration	TNT-1 F(ab′)2 ME-180	^45^
Histamine conjugate	Enhanced vasopermeability	118% increase in uptake 3 days after administration	TNT-1 F(ab′)2 ME-180	^45^
Physalaemin conjugate	Enhanced blood flow	71% increase in uptake 3 days after administration	TNT-1 F(ab′)2 ME-180	^45^
Bradykinin conjugate	Enhanced blood flow	23% increase in uptake 3 days after administration	TNT-1 F(ab′)2 ME-180	^45^
PEP2-Ab conjugate	Enhanced vasopermeability	249% increase in uptake 3 days after administration	B72.3 LS174T	^46^
A22p-Ab conjugate	Enhanced vasopermeability	50%–100% increase in uptake at 3 and 12 h after administration	Cetuximab/A459 Trastuzumab/SKOV3	^49^
iRGD	Enhanced vasopermeabiltiy	3,900% Increase at 3 h by ELISA	Trastuzumab BT-474	^48^
Mannitol Infusion	Osmotic opening of BBB	234% and 32% increase in F(ab′)2 and mAb AUC (0.5–72 h)	P1.17 LX-1 SCLC	^58^
Focused ultrasound	Transient disruption of BBB	5,577% increase in uptake 2 h after administration	Bevacizumab U87 glioma	^63^
Angiopep-2-Ab conjugate	RMT through BBB by LRP1 binding	˜300% increase in tumor uptake 24 h after administration	Trastuzumab BT-474	^68^
Human melanotransferrin-Ab conjugate	RMT through BBB by LRP1 binding	415% increase in tumor uptake 2 h after administration	Trastuzumab MDA-MB-231-BR^HER2/eGFP^	^69^

To increase mAb tumor uptake, many preclinical investigations have explored the use of agents that enhance vascular permeability and blood flow. Early work by several groups reported co-administration of mAb with the vasculature promoting agents: angiotensin II, tumor necrosis factor-alpha, interferon, and interleukin 2 led to between 40% and 200% increases in mAb tumor uptake^41–44^. The utility of these agents was limited by the short plasma half-life of the signaling ligands and the lack of tumor specificity of the vascular permeability enhancement leading to increased mAb deposition in healthy organs^41,43,44^. To overcome these limitations, the Epstein group made chemical conjugates of 7 vascular promoting agents with the anti-necrosis mAb TNT-1 F(ab′)2^45^. A TNT-1 F(ab′)2 conjugate with interleukin 2 led to the greatest enhancement in the tumor uptake of ^125^I-TNT-1 (Fab′), with a 275% increase in tumor radioactivity 3 days after administration^45^. The domain of interleukin 2 responsible for increasing vasculature permeability was isolated to a 37 amino acid sequence termed permeability-enhancing peptide (PEP)^46^. Administration of mAb-PEP conjugates, 2 h prior to administration of ^125^I-mAb, increased the uptake of TNT-1 and Lym-1 mAbs by 4-fold, 3 days after administration^46^. The neuropilin-1 receptor, which, upon ligand binding, accumulates at the inter-endothelial cell contacts and induces vascular permeability, is the target for 2 peptides that have been reported to increase mAb tumor uptake^47^. Sugahara et al.^48^ developed a cyclic 9mer peptide, named iRGD, that binds αν-integrins that are expressed on tumor endothelium. An internal cleavage sequence results in proteolytic digestion of the iRGD peptide and αν-integrin dissociation^48^. The cleaved iRGD peptide has a CendR motif that binds to neuropilin-1 and, as a result, increases vasculature permeability^48^. Co-administration of 4 μmol/kg iRGD with 3 mg/kg trastuzumab to mice bearing orthotopic BT474 xenografts increased trastuzumab uptake by 40-fold, 3 h after administration^48^. Shin et al.^49^ reported a peptide that was derived from the neuropilin-1 ligand semaphorin 3A, that was modified for enhanced neuropilin-1 binding affinity, named A22p. Genetic fusion of the A22p peptide to the carboxy terminus of the Fc domain of cetuximab or trastuzumab increased mAb tumor uptake between 1.5- and 2-fold at 3 and 12 h post-administration^49^. Both the iRGD peptide and A22p peptide were reported to significantly improve the preclinical efficacy of trastuzumab^48,49^. To our knowledge, there are no clinical trials involving any of the listed blood flow/permeability enhancers in combination with approved anti-cancer mAbs. The iRGD peptide is currently being evaluated in combination with nab-paclitaxel and gemcitabine for metastatic pancreatic cancer in a phase II trial (registration No. NCT03517176).

The effective targeting of brain tumors with mAb-based therapies is hampered by the blood–brain barrier (BBB)^50^. The BBB is highly selective and limits the extravasation of mAbs through the brain vasculature and into the brain interstitial fluid^51,52^. Using a combination of microdialysis and tissue ELISA, Chang et al.^53^ reported that the concentration of trastuzumab in rat brains, following systemic administration, was between 377- and 909-fold less than the concentration of trastuzumab in plasma. Wang et al.^54^ reported similar cerebrospinal fluid concentrations of 5 humanized mAbs in rats and cynomolgus monkeys with a cerebrospinal fluid/plasma ratio of 0.1%–0.2%. Contrast-enhanced magnetic resonance imaging of brain lesions indicates there is a partial disruption of the BBB in tumors; however, this breakdown is variable between tumor subtypes and heterogenous within individual tumors^55^. In patients with metastatic breast cancer, the concentrations of ^89^Zr-trastuzumab were observed to be 17-fold greater in brain lesions than healthy brain tissue^56^; however, this concentration enhancement may also be the result of higher HER2 antigen expression in tumors relative to healthy brain tissues. Administration of ^89^Zr-bevacizumab to children with diffuse intrinsic pontine glioma demonstrated the variability in brain tumor uptake of antibody therapy, with 5 out of 7 patients showing detectable tumor uptake, at 144 h post-injection, with standardized uptake ratios varying between 1.0 and 6.7^57^. Manual disruption of the BBB to enhance mAb uptake in brain tumors has been evaluated using several methods. Intracarotid infusion of mannitol results in an osmotic opening of the BBB and has been reported to enhance tumor uptake of intact mAb and Fab/F(ab′)2 fragments in rats bearing intracerebral lung carcinoma xenografts^58^. Modulation of calcium-dependent potassium channels with the channel agonist NS-1619 enhances brain tumor vasculature permeability^59^, with NS-1619 co-administration increasing trastuzumab tumor uptake in a mouse xenograft glioma model^60^. Focused ultrasound (FUS) has been reported to enhance mAb delivery to brain tumors through transient modulation of the BBB. Kinoshita et al.^61^ reported that FUS increased the uptake of trastuzumab into mouse brains from below the limit of quantification (780 ng/g of tissue) to 3,257 ng/g. Combining FUS with trastuzumab increased the median survival of *nu*/*nu* rats bearing BT474 brain xenografts by greater than 32%^62^. The combination of FUS with bevacizumab resulted in a 5.7- to 56.7-fold increase in bevacizumab brain concentrations and significantly improved the therapeutic effect of bevacizumab in mice bearing U87 brain xenografts in comparison to bevacizumab alone (mean survival time of 73 *vs.* 46 days)^63^. Brighi et al.^64^ reported that FUS significantly increased the uptake of the anti-EphA2 mAb 4B3 in a patient derived xenograft mouse model of high-grade glioma; however, significant increases were only observed in the non-contrast-enhancing tumors (indicative of tumors with a functional BBB). There are 4 clinical trials evaluating FUS in combination with traditional chemotherapies (registration Nos. NCT03712293, NCT02343991, NCT03322813, and NCT03616860)^64^; however, to our knowledge there are no clinical trials evaluating FUS with mAb therapies. Significant efforts have been placed into development of antibody conjugates that can bind to receptors that are expressed on the BBB to allow brain uptake through receptor-mediated transcytosis (RMT). The transferrin receptor (TfR) and insulin receptor (IR) are common targets for antibody RMT^65–67^. To our knowledge, the impact of TfR/IR binding on therapeutic antibody uptake into brain tumors has not been reported. An additional receptor of interest for RMT is the low-density lipoprotein-like receptor 1 (LRP1). Conjugation of the peptide angiopep-2, which binds to LRP1, resulted in a 6-fold increase in brain/serum ratios for an anti-HER2 antibody and increased the median survival of mice bearing BT474 xenografts by 20%, relative to unconjugated antibody^68^. Conjugation of melanotransferrin, a substrate of LRP1, to trastuzumab resulted in a 10- to 225-fold increase in the brain/blood concentration ratio of trastuzumab and reduced the size and number of metastatic brain tumors in mice administered MDA-MB-231-BR^HER2/eGFP^ breast cancer cells^69^.

### Extracellular matrix

The hyperpermeability of solid tumor vasculature results in the deposition of the plasma proteins fibrin and fibrinogen into the interstitial space of solid tumors^70^. Fibrinogen forms a scaffold that binds inflammatory factors and recruits macrophages and fibroblasts^70^. Over time, the recruited inflammatory cells form a mature matrix^70^, which is, in part, composed of collagen and hyaluronan. Hyaluronan is a negatively charged glycosaminoglycan that causes electromechanical repulsion and water absorption, leading to tumor swelling^71^. Highly crosslinked collagen fibers resist the tumor swelling induced by hyaluronan and cell proliferation^71^, which causes tensile stress that collapses tumor vasculature and lymphatics, contributing to the high tumor IFP^71^. Post-extravasation, the dense extracellular matrix slows mAb diffusion, limiting tumor penetration^72–77^. The limiting effect of the tumor extracellular matrix on mAb uptake and penetration in solid tumors is well appreciated^20–22^; as a result, significant effort has been dedicated to developing methods that overcome tumor matrix barriers and enhance mAb tumor uptake and penetration (**Table 3**).

**Table 3:** Extracellular matrix modulation approaches

Treatment	Impact on tumor	Impact on mAb tumor PK	mAb/tumor model	Source
Intratumoral collagenase	Collagen degradation	80%–100% increase in diffusion 24 h after collagenase injection	Non-specific IgG (S1) HSTS-26T and U87	^77^
Intravenous collagenase	Collagen degradation/decrease in tumor interstitial fluid pressure	90%–140% increase in uptake at 24 h	TP-3 OHS	^79^
Relaxin infusion	Downregulation of tumor fibrosis/decrease collagen fiber length and signal	80% increase in diffusion coefficient after a 12-day relaxin infusion	Non-specific IgG HSTS-26T	^78^
Intratumoral injection of bovine hyaluronidase	Hyaluronan degradation/decrease in interstitial fluid pressure	70% increase in uptake 9 days after administration	TP-3 OHS	^80^
Pegylated human hyaluronidase (PEGPH20)	Tumor hyaluronan degradation	100% increase in uptake 2 days after administration	Trastuzumab SKOV3-HAS2	^82^
Pulsed ultrasound	Structural modification of extracellular matrix and widening of intercellular gaps	36% Increase in tumor AUC (0–5 days)	MX-B3 A431	^130^

Significant contributions to understanding the importance of the tumor stroma on mAb transport have been made by the Jain group^22,72,77,78^. Using fluorescence recovery after photobleaching, Netti et al.^77^ observed that the rate of immunoglobulin G (IgG) diffusion in xenografts with high tumor collagen content (˜6–8 mg/g) was 2-fold slower than that of IgG diffusion in tumors with low collagen content (˜1.5 mg/g). Peri-tumoral injection of 0.3 mL of 10% *Clostridium* collagenase increased the diffusion coefficient of a non-specific IgG in the xenografts with high collagen content by 2-fold^77^. Eikenes et al.^79^ reported that the intravenous injection of 100 μg of *Clostridium* collagenase into mice bearing OH3 xenografts decreased the mean venous pressure and tumor IFP by 60% and 45%, respectively. The mean venous pressure returned to baseline 80 min after injection, whereas the tumor IFP reached a nadir at 6–7 h post-dosing. The decrease in tumor IFP with collagenase administration enhanced the tumor uptake of the mAb TP-3 by 2-fold^79^. Many additional preclinical studies have demonstrated the beneficial impact of collagenase on the tumor uptake of co-administered therapies^73^. Despite the preclinical benefits that have been observed with collagenase co-therapy, off-target toxicities and concern of increased tumor metastasis following collagenase administration have precluded clinical translation^73^.

Hyaluronan degradation with hyaluronidase has also been explored for improving co-administered mAb uptake. Intra-tumoral injection of bovine hyaluronidase to mice bearing OHS xenografts increased the uptake of the TP3 mAb by 70% 9 days after dosing^80^. To overcome the short plasma half-life of hyaluronidase (˜3 min), Halozyme developed a PEGylated recombinant human hyaluronidase (PEGPH20) with a 10-h plasma half-life^81^. Administration of 40 μg/kg of PEGPH20 to mice bearing SKOV3/HAS2 xenografts increased AlexaFluor488-trastuzumab uptake by 2-fold 48 h after injection^82^. PEGPH20 recently failed to reach the primary endpoint in a phase III clinical trial in combination with gemcitabine and nab-paclitaxel for metastatic pancreatic cancer (NCT02715804). A potential contribution to the failure is dose-limiting toxicities in the phase I trial that limited the PEGPH20 dose to 3 μg/kg, a fraction of the preclinical doses used to improve mAb/chemotherapeutic uptake^81–84^.

Fibrosis pathway antagonists that can limit matrix deposition in solid tumors have drawn a significant amount of interest. The peptide hormone relaxin-2 decreases transforming growth factor-beta (TGF-β)–mediated fibrosis after binding its cognate receptor relaxin family peptide receptor 1^85,86^. Brown et al.^78^ used second harmonic generation imaging to evaluate the impact of a 12-day infusion of relaxin on tumor collagen in mice bearing HSTS26T xenografts in dorsal skinfold chambers. Relaxin infusion decreased the length of tumor collagen fibers and decreased the signal of preexisting collagen fibers, resulting in an 80% increase in the diffusion coefficient of a non-specific IgG. Intra-tumoral expression of relaxin using genetically modified hematopoietic stem cells increased trastuzumab efficacy in mice bearing BT474-M1 and HCC1954 xenografts^87^. Currently, there is an interest in using relaxin as a therapy in many fibrotic diseases^88–90^. However, relaxin recently failed to reach its primary endpoint in a phase III clinical trial for acute heart failure^91^. The clinical success of relaxin may be limited due to its short plasma half-life^92^, and several groups have developed lipid and Fc conjugates with relaxin to extend the persistence of exposure in plasma^93,94^. An additional concern that may have precluded clinical pursuit of relaxin for anti-cancer treatment is the observation that relaxin signaling is involved in tumor progression and metastasis in several cancers^95,96^. However, a recent report demonstrated intratumoral relaxin expression decreased metastasis in 4 murine metastatic models, and synergistically increased the efficacy of an anti-PD-L1 fusion protein^97^. There are many other fibrosis pathway antagonists that have been explored both in the clinic and preclinically, including the angiotensin inhibitor losartan^98^, sonic-hedgehog antagonists^99^, and TGF-β inhibitors^100,101^; however, to our knowledge, the impact of these therapies on co-administered mAb uptake has not been reported. Losartan is currently in a phase II clinical trial in combination with nivolumab and the chemotherapeutic regime FOLFIRINOX for the treatment of localized pancreatic cancer (registration No. NCT03563248).

### Antigen and cellular barriers

Tumor-associated cells can limit mAb tumor uptake and penetration in many ways. The rapid proliferation of cancer cells causes growth-induced solid stress that contributes to the collapse of blood and lymph vessels^102,103^. Antigen-expressing cells can also act as catabolic sinks, where a small number of tumor cells that surround vasculature can bind to and eliminate a significant portion of extravasated mAb^26,104,105^. Antigen and cellular barriers can act synergistically with tumor vasculature and matrix barriers to decrease mAb tumor uptake and penetration; as a result, approaches to modulate antigen and cellular barriers have been pursued (**Table 4**).

**Table 4:** Antigen and cellular modulation approaches

Approach	Impact on mAb tumor PK	mAb/tumor model	Source
pH sensitive mAb	30% increase in tumor AUC (0–14 days) in comparison to non-pH sensitive mAb T84.66	10H6/T84.66 MC38CEA+	^126^
Paclitaxel administered 2 days after mAb administration	30% increase in cumulated activity 0–6 days	^111^In-DOTA-Gly3Phe-m170/breast or prostate cancer	^106^
5-fluorouracil treatment 2 days prior to mAb administration	148% increase in uptake 5 days after administration	^125^I-NHS76 Colon 26	^107^
Paclitaxel treatment 2 days prior to mAb administration	102% increase in uptake 5 days after administration	^125^I-NHS76 Colon 26	^107^
Tofacitinib	48% increase (LMB) and 133% increase (BV421) in tumor cell uptake/binding 3 h after administration	LMB-100/KLM-1 BV421/MDA-MB-461	^108^
Junction opener 1	500% increase in tumor uptake 12 h after administration	Trastuzumab HCC1954	^109^

The most clinically relevant approach to overcome cellular barriers to mAb tumor uptake and penetration is the co-administration of traditional chemotherapeutics. In fact, many of the FDA-approved mAb therapies are approved in combination with chemotherapy (**Table 1**). In a small phase I clinical trial, the mAb ^111^In-Gly3Phem170 was administered to patients with breast or prostate cancer on 2 occasions, 1 week apart, and the tumor-accumulated activity was evaluated^106^. One group in the trial received an infusion of paclitaxel 2 days after the second mAb dose. No change in mAb uptake was observed in the control group, whereas a 30% increase in tumor radioactivity was observed between the first and second doses for patients who received paclitaxel^106^. The trough concentrations of trastuzumab in patients who receive paclitaxel were observed to be 1.5-fold higher than those of patients in whom only trastuzumab was administered. The increase in trastuzumab trough concentrations may be the result of paclitaxel killing HER2-expressing tumor cells, decreasing target mediated disposition. In a preclinical study, the co-administration of the chemotherapeutics 5-fluorouracil, etoposide, vinblastine, paclitaxel, or doxorubicin increased the tumor uptake of the radiolabeled tumor necrosis targeting mAbs chTNT-3 and NHS76^107^. Recently, tumor-associated inflammatory cells have been implicated as a non-specific elimination pathway for extravasated mAb^108^. Administration of the janus kinase (JAK) inhibitor tofacitinib to xenograft bearing mice decreased the number of tumor-associated monocytes, macrophages, and neutrophils^108^. Co-administration of tofacitinib with the immunotoxin LMB-100 or the anti-epidermal growth factor receptor (EGFR) mAb BV421 increased the number of tumor cells with LMB-100 uptake by 48% and the number of tumor cells with BV421 uptake by 133%, 3 h after administration^108^. The same study reported tofacitinib co-administration improved the efficacy of LMB-100 and anetumab ravtansine in xenograft bearing mice^108^. Tumor cells also form tight intercellular junctions that significantly impede antibody penetration^109^. Beyer et al.^109^ isolated a small protein named junction opener 1, which decreased the formation of tight junctions between tumor epithelial cells. Administration of 2 mg/kg of junction opener 1 to mice bearing HCC1954 xenografts, 1 h prior to the administration of 10 mg/kg trastuzumab, resulted in a 500% increase in trastuzumab tumor uptake at 12 h^109^. The same study reported junction opener 1 increased the efficacy of trastuzumab against BT474-M1, HCC1954, NCI-N87, and SKOV3 xenografts and increased the efficacy of cetuximab against A549 xenografts^109^.

Cellular antigen kinetics can unfavorably alter mAb disposition and has been implicated as a cellular resistance pathway for mAb therapy. Many tumor-associated antigens, including HER2, CEA, mesothelin, and EGFR, undergo proteolysis that results in the release of soluble antigen (often referred to as shed antigen)^110,111^. Our group observed a 2-fold increase in T84.66 plasma clearance with coadministration of soluble CEA, resulting in a 55% decrease in T84.66 uptake into LS174T xenografts^112^. The tumor uptake of the zirconium-89 (^89^Zr)–labeled anti-EGFR mAb imgatuzumab was significantly decreased in mice bearing xenografts of the EGFR shedding cell-line A431 in comparison to the uptake in A549/H441 xenografts with low-EGFR shedding rates^113^. A significant increase in the liver uptake of ^89^Zr-imgatuzumab was observed in mice bearing A431 xenografts, indicating plasma shed antigen can act as an antibody sink through enhanced non-specific elimination^113^. Iwano et al.^114^ developed an anti-CEA mAb, 15-1-32, with preferential binding for membrane CEA. Co-administration of soluble CEA with 15-1-32 decreased the plasma AUC by 22.5%, whereas the plasma AUC of a non-preferential anti-CEA mAb labetuzumab was decreased by 79.9%^114^. Shed mesothelin has also been reported to decrease immunotoxin efficacy^115^. Co-administration of paclitaxel with the immunotoxin SS1P to KB-3-1 xenograft–bearing mice, synergistically increased the anti-tumor effect, which was attributed to a decrease in tumor shed antigen concentrations^116^.

Counterintuitively, high cellular antigen expression has been implicated as a primary barrier to mAb efficacy. High antigen expression and rapid antigen internalization, combined with the slow tumor uptake and the slow interstitial diffusion of therapeutic mAb, result in poor mAb penetration. The limiting effect of antibody binding to tumor antigens on antibody distribution within solid tumors is commonly referred to as the “binding site barrier”^26,104,117^. As a result of the binding site barrier, at sub-saturating doses, mAb appears trapped around tumor vasculature^118–120^. For mAb therapies that can efficiently kill tumor cells with only a fraction of cellular antigen bound, such as antibody–drug conjugates, the binding site barrier results in sub-optimal tumor exposure, decreasing efficacy^121–123^. Approaches to improve mAb penetration include the use of antibody fragments (Fabs, scFvs, VHHs) or low-affinity mAbs. Antibody fragments can diffuse through the interstitial space more rapidly than intact mAbs, increasing tumor penetration prior to binding. The rapid plasma elimination of antibody fragments requires high fragment doses to saturate tumor antigen, and high binding affinity is required to retain antigen-binding once plasma concentrations drop below tumor concentrations^26,124^. However, high-affinity single-chain variable fragments (scFvs) have been shown to have restricted tumor distribution similar to intact mAb^125^, and fragments are unable to recruit immune effector cells. Low-affinity mAbs can diffuse a greater distance from sites of extravasation prior to antigen binding and can undergo multiple dissociation–association steps prior to receptor-mediated internalization and elimination^26,104^. However, the effective saturation of tumor antigen with a low-affinity mAb requires high mAb doses, decreasing tumor selectivity^26,104^. Our laboratory developed a pH-sensitive anti-CEA mAb, 10H6, that efficiently detaches from internalized CEA following endosomal acidification^126^. Following antigen dissociation, the pH-sensitive mAb is recycled by neonatal Fc receptor (FcRn) with transport to the interstitial space, allowing 10H6 to undergo multiple CEA binding and internalization events prior to elimination^126^. In comparison to the non-pH sensitive anti-CEA mAb T84.66, 10H6 had a higher tumor and plasma AUC in mice bearing the human CEA expressing mouse cell line MC38^126^. No difference in tumor AUC or plasma AUC was observed for mice bearing the human cell-line LS17T4, as 10H6 is a murine mAb and is unable to bind the human FcRn that is expressed in LS174T cells. These results indicate that, following humanization, 10H6, and other pH-sensitive mAbs, can be used, in part, to increase the tumor retention and tumor penetration of mAb^126^. Additionally, it is likely 10H6 is less sensitive to shed CEA-mediated elimination, as 10H6 that binds shed CEA will dissociate and undergo FcRn recycling following non-specific uptake into elimination organs.

## Conclusions

The dramatic growth of mAb therapies over the past decade has resulted in mAbs becoming one of the most important anti-cancer therapies. Despite the continued growth of mAb therapies, the physiology of solid tumors has significantly limited the efficacy of anti-cancer mAbs. In this review, we have discussed many of the preclinical methods that have been reported to improve mAb tumor uptake and distribution. Many of the early methods relied on co-administered proteins to enhance vasculature permeability or degrade tumor matrix; however, these strategies failed to translate clinically due to poor selectivity. Some of the recent preclinical strategies that are reported to enhance mAb tumor disposition, including tumor-selective vasculature permeability enhancers and pH-sensitive mAbs, may achieve clinical translation in the next several years. Additionally, SMDs that have been shown to impact solid tumor physiology and are approved for other indications, such as losartan or tofacitinib, may be rapidly repurposed to increase mAb efficacy. Also, although not discussed in the present review, simple physical treatments, including hyperthermia^127–129^ or application of ultrasound^130^, have also been demonstrated to improve solid tumor uptake of mAb and may be facilely implemented into clinical protocols. As methods to increase the tumor uptake and distribution of mAb become more selective and more effective, it is likely that for solid tumor therapy, mAbs will soon live up to the nickname “magic bullets”.
